# ［2+4］型手性多孔有机笼Pim高效液相色谱固定相的制备及其手性拆分性能评价

**DOI:** 10.3724/SP.J.1123.2026.01017

**Published:** 2026-10-08

**Authors:** Chun CHEN, Yanan GONG, Bangjin WANG, Shengming XIE, Junhui ZHANG, Liming YUAN

**Affiliations:** 云南师范大学化学化工学院，云南省现代分离分析与物质转化重点实验室，云南 昆明 650500; Yunnan Key Laboratory of Modern Separation Analysis and Substance Transformation，College of Chemistry and Chemical Engineering，Yunnan Normal University，Kunming 650500，China

**Keywords:** 高效液相色谱, 手性固定相, 对映体分离, 手性多孔有机笼, high performance liquid chromatography （HPLC）, chiral stationary phase （CSP）, enantioseparation, chiral porous organic cage

## Abstract

多孔有机笼（POCs）是一类由分立的三维笼状分子通过分子间作用组装形成的新型多孔材料，具有良好的溶解性和独特的多孔结构，在气体吸附、分子识别、分离、催化、传感等领域展现出良好的应用前景。本文以酚酞为底物，三氟乙酸和六次甲基四胺为甲酰化试剂，由Duff反应合成了基于酚酞的四醛基化合物，再与（1*R*，2*R*）-（-）-1，2-环己二胺通过亚胺缩合反应合成了一种基于酚酞的［2+4］型手性亚胺多孔有机笼（Pim， C_72_H_68_N_8_O_8_）。将合成的Pim用5-溴-1-戊烯修饰烯基功能化后与巯基硅胶通过巯基-烯点击反应制得了一种新型高效液相色谱（HPLC）手性固定相（CSP），并评价了其在正相高效液相色谱（NP-HPLC）和反相高效液相色谱（RP-HPLC）分离模式下的手性拆分性能。结果表明，该CSP填充的Pim柱在NP-HPLC和RP-HPLC分离模式下均表现出良好的手性分离性能。其中，在NP-HPLC分离模式下成功拆分了10种外消旋体，在RP-HPLC分离模式下成功拆分了9种外消旋体。所制备的Pim柱与两种广泛使用的Chiralpak AD-H和Chiralcel OD-H商品柱具有良好的手性拆分互补性，能拆分这两种商品柱不能拆分或拆分效果不理想的一些手性化合物。此外，所制备的Pim柱还具有良好的分离重复性和稳定性。历经数百次进样使用后，其对外消旋体拆分的保留时间和分离度与柱使用之初相比无明显变化，相对标准偏差（RSD，*n*=5）分别≤1.50%和≤1.92%；在柱放置保存1个月后，其分离效果与柱使用之初的分离效果也无显著改变，保留时间和分离度的RSD（*n*=3）分别≤1.88%和≤2.26%。本研究证明手性POC Pim是一种良好的HPLC手性分离材料，为基于POCs的新型HPLC手性固定相的开发提供了重要的理论与实验依据，也对拓展POCs类CSPs具有重要意义。

## Abstract (en)

Porous organic cages （POCs） represent a class of emerging porous materials assembled from discrete three-dimensional cage-like molecules via intermolecular interactions. Owing to their good solubility and unique pore structures， they show great potential in gas adsorption， molecular recognition， separation， catalysis， and sensing. In this study， a phenolphthalein-based tetraaldehyde compound was synthesized via the Duff reaction using phenolphthalein as the substrate and trifluoroacetic acid （TFA） combined with hexamethylenetetramine （HMTA） as the formylation reagent. Subsequently， a ［2+4］-type chiral imine POC， Pim （C_72_H_68_N_8_O_8_）， was constructed by reacting the tetraaldehyde precursor with （1*R*，2*R*）-（-）-1，2-diaminocyclohexane via imine condensation. The synthesized Pim was functionalized with alkenyl groups using 5-bromo-1-pentene and then immobilized onto thiol-modified silica via thiol-ene click chemistry to afford a novel chiral stationary phase （CSP） for high performance liquid chromatography （HPLC）. The successful syntheses of tetraaldehyde compound， Pim， and CSP were characterized by nuclear magnetic resonance （NMR）， fourier transform infrared spectroscopy （FT-IR）， electrospray ionization mass spectrometry （ESI-MS）， thermogravimetric analysis （TGA）， and elemental analysis （EA）. The chiral separation performance of this CSP was evaluated under both normal-phase （NP） and reversed-phase （RP） HPLC separation modes. The results demonstrated that the CSP exhibits excellent chiral separation capabilities in both two separation modes. Under NP-HPLC conditions， ten racemates were resolved， including five with baseline separation. Under RP-HPLC conditions， nine racemates were resolved， of which eight achieved baseline separation. Furthermore， this CSP exhibits good chiral separation complementarity with two widely used commercial columns， Chiralpak AD-H and Chiralcel OD-H， showing the ability to resolve some chiral compounds that cannot be resolved or are poorly resolved by the two commercial columns. The effects of analyte injection mass and column temperature on chiral separation performance of the column were also investigated. Additionally， the prepared HPLC column exhibits good repeatability and stability. After hundreds of injections， no significant changes were observed in the retention times and resolutions compared to its initial performance， with relative standard deviations （RSDs， *n*=5） of ≤1.50% and ≤1.92%， respectively. Meanwhile， after the column was placed and stored for one month， its separation performance showed no significant change compared with that at the initial stage of column use， and the RSDs （*n*=3） of retention time and resolution were ≤1.88% and ≤2.26%， respectively. This study confirms that the chiral POC Pim is a promising material for HPLC chiral separation， providing significant theoretical and experimental support for the development of novel POC-based CSPs and holding great significance for expanding the POC-based CSPs.

由于同一手性化合物的两个对映体在生物体内的药理活性、代谢途径及毒性等方面可能表现出显著差异，甚至会表现出完全相反的作用效果，因此通过手性分离获取单一构型对映体在生物医药、有机合成、农业化学、食品科学等领域具有重要的意义^［1-3］^。在当前应用的各种手性分离方法中，基于手性固定相（CSP）的高效液相色谱（HPLC）法因其具有分离效率高和适用性广等优点，被公认为是手性分离的主流方法之一^［4，5］^。CSP的综合性能直接决定了手性分离的选择性与分离效率，因此研究高性能新型CSP也是该方法的研究核心，并引起了极大的研究兴趣。近年来，以金属有机框架（MOFs）^［6-9］^、共价有机框架（COFs）^［10-13］^及微孔有机网络聚合物（MONs）^［14］^为代表的多孔材料凭借其具有的高比表面积、明确规整的多孔结构以及优良的主-客体识别特性，被广泛用作固定相用于色谱分离研究。

多孔有机笼（POCs）是由两种及以上有机构筑单元经亚胺缩合或硼酸酯缩合或烯/炔烃复分解等反应合成的分立三维笼状分子通过分子间作用力组装形成的一类新型多孔固体材料^［15-17］^。与MOFs、COFs、MONs等多孔框架材料相比，POCs是借助较弱的分子间作用力而非强配位键或共价键将分立的笼状分子组装形成多孔固体，其典型特点是具有良好的溶解性，因此POCs溶解形成溶液后更易于结构修饰、化学键合、制备多孔复合材料、涂敷成膜等^［18］^。另外，POCs不但具有笼状分子的分子内固有空腔，而且分立的笼分子间不紧密的组装还能形成多样的外部堆积孔隙结构。由于POCs的这些特性，近些年来其在气体吸附^［19，20］^、分子识别^［21，22］^、分离^［23］^、催化^［24］^、荧光传感^［25］^、生物医药^［26］^等领域被广泛研究和应用。POCs也是一类极具潜力的色谱分离材料。2015年，本课题组将一种手性POC（CC3-R）作为固定相涂覆制备了毛细管气相色谱（GC）柱，实现了50多种外消旋体的高效拆分^［27］^，并与商品β-DEX 120毛细管GC柱进行了对比，可拆分β-DEX 120柱不能拆分的许多外消旋体，表明其优异的GC手性拆分性能和良好的应用前景。随后，一些不同结构和孔径的手性POCs（如CC9、CC10、NC1-R等）也相继被研究作为GC手性固定相^［28-31］^，并展现出优秀的手性拆分性能。相比之下，POCs在HPLC中的应用研究相对较晚，其原因是POCs具有良好的溶解性和低骨架密度，通常需要化学键合于硅胶基质上制备HPLC手性固定相，而合成含-OH、-NH_2_、-COOH等可用于化学修饰或键合的活性官能团的手性POCs存在很大挑战性。直到2022年，我们将一种含-OH官能团的手性POC NC1-R通过化学键合于巯基硅胶上制备了CSP用于HPLC手性分离，证实了POCs作为HPLC手性固定相的潜在应用价值^［32］^。此后，RCC2-S^［33］^、CC19-R^［34］^、CC3-R^［35，36］^等手性POCs也被研究用于HPLC手性分离。然而，目前将手性POCs用于HPLC手性分离的研究仍然有限。研究具有不同手性识别位点、空腔微环境及拓扑结构的手性POCs用于HPLC手性分离，对丰富和发展基于POCs的新型HPLC手性固定相仍然具有重要的科学意义与应用价值。

本研究首先以酚酞作为反应底物，三氟乙酸和六次甲基四胺为甲酰化试剂，经Duff反应合成了一种基于酚酞的四醛基化合物。随后，将该四醛基化合物与（1*R*，2*R*）-（-）-1，2-环己二胺通过亚胺缩合反应制备得到了基于酚酞的［2+4］型手性亚胺POC Pim。将合成的Pim烯基功能化修饰后通过巯基-烯点击反应策略键合在巯基化硅胶表面制备了HPLC手性固定相，评价了其在正相高效液相色谱（NP-HPLC）和反相高效液相色谱（RP-HPLC）分离模式下对外消旋体的手性拆分性能，并与商品Chiralcel OD-H和Chiralpak AD-H手性柱进行了对比。除此之外，还探讨了柱温和进样量对手性分离效果的影响，并评价了柱的重复性和稳定性。

## 1 实验部分

### 1.1 仪器、试剂和材料

LC-16高效液相色谱仪（日本Shimadzu公司）；1666型高效液相色谱装柱机（美国Alltech公司）；Avance NEO 500 MHz核磁共振波谱仪（NMR，德国Bruker公司）；Nicolet iS10傅里叶红外光谱仪（FT-IR）、LTQ-Orbitrap XL电喷雾电离质谱仪（ESI-MS，美国Thermo Fisher公司）；SDT-650热重分析仪（TGA，美国Taber公司）；Vario EL Ⅲ元素分析仪（EA，德国Elementar公司）。

酚酞（纯度≥99%）购自上海Aladdin生化科技股份有限公司；5-溴-1-戊烯（纯度≥99%）、（3-巯基丙基）三甲氧基硅烷（纯度≥98%）、偶氮二异丁腈（AIBN，纯度≥98%）、三乙胺（纯度≥99%）、氢化钠（NaH，纯度60%）、六次甲基四胺（HMTA，纯度≥99%）、（1*R*，2*R*）-（-）-1，2-环己二胺（纯度≥99%）和三氟乙酸（TFA，纯度≥99%）购自上海Adamas-beta试剂公司；盐酸（纯度≥36%）、甲苯（纯度≥99.5%）、吡啶（纯度≥99%）、乙醇（EtOH，纯度≥99%）、二氯甲烷（CH_2_Cl_2_，纯度≥99.5%）、丙酮（纯度≥99.5%）和三氯甲烷（CHCl_3_，纯度≥99%）购自天津风船化学试剂科技有限公司；HPLC级正己烷（*n*-HEX）、异丙醇（2-PrOH）、甲醇（MeOH）和乙腈（MeCN）购自上海Sigma-Aldrich公司；外消旋体（纯度≥98%）购自上海Adamas-beta试剂公司、Sigma-Aldrich公司、TCI公司及Aladdin生化科技股份有限公司；硅胶（5 µm，比表面积300 m^2^/g）购自苏州纳微科技有限公司；Chiralpak AD-H色谱柱（250 mm×4.6 mm）和Chiralcel OD-H色谱柱（250 mm×4.6 mm）购自上海大赛璐药物手性技术有限公司；HPLC空柱（250 mm×2.1 mm）购自大连日普利科技仪器有限公司。

### 1.2 实验过程

#### 1.2.1 ［2+4］型手性POC Pim的合成

［2+4］型手性POC Pim的合成参考文献［^37^］，合成流程见图1。在氮气保护下，向25 mL TFA溶液中加入酚酞（2.38 g，7.48 mmol）和HMTA（15.75 g，11.22 mmol），随后将反应体系升温至100 ℃，搅拌回流反应48 h。反应结束后，待体系冷却至室温，向其中缓慢加入150 mL浓度为4 mol/L的盐酸溶液，继续在100 ℃条件下回流2 h。冷却至室温后，采用真空抽滤对反应混合物进行分离，得到黄色固体粉末，通过柱色谱（CH_2_Cl_2_-MeOH，10:1，体积比）纯化获得基于酚酞的四醛基化合物1。

**图1:**
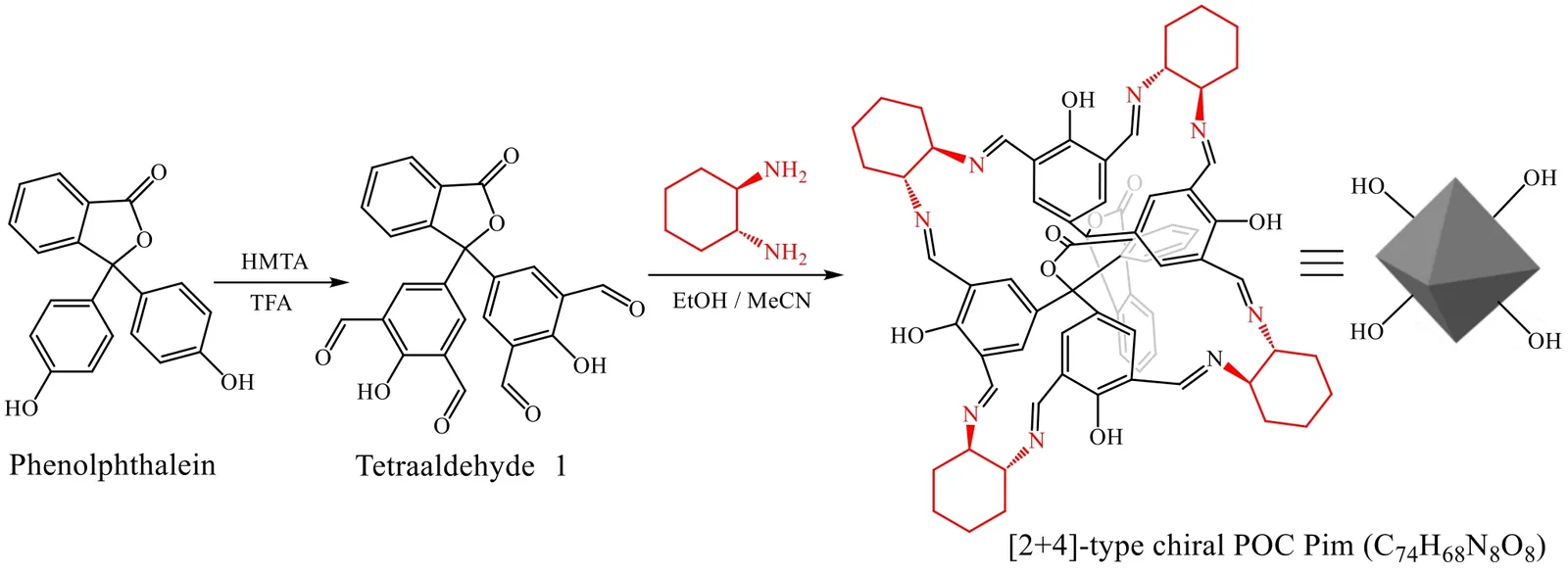
［2+4］型手性POC Pim的合成

将上述制得的四醛基化合物1（0.30 g，0.70 mmol）溶解于150 mL MeCN中，另取（1*R*，2*R*）-（-）-1，2-环己二胺（0.16 g，1.40 mmol）溶解于60 mL EtOH中。将这两种溶液充分混合后，在N_2_保护下70 ℃回流反应24 h。反应结束后冷却至室温，通过过滤收集反应生成的黄色固体产物，并用MeCN洗涤，干燥，最终得黄色固体为［2+4］型手性POC Pim。

#### 1.2.2 CSP的制备

通过巯基-烯点击反应将所合成的［2+4］型手性POC Pim化学键合于巯基化硅胶表面制备高效液相色谱CSP，具体制备路线如图2所示。首先参照文献［^34^，^38^］报道的方法制备巯基化硅胶（SiO_2_-SH）。然后，用5-溴-1-戊烯对手性Pim进行烯基功能化，具体过程如下：在N_2_保护下，向100 mL双颈圆底烧瓶中依次加入NaH（0.6 g）和30 mL无水CHCl_3_，并将烧瓶置于0 ℃冰浴中搅拌20 min。通过恒压滴液漏斗逐滴加入20 mL预先溶解有手性Pim（0.62 g，0.53 mmol）的无水CHCl_3_溶液，滴加完毕后继续在0 ℃冰浴下搅拌30 min，再加入1.4 mL 5-溴-1-戊烯，体系升温至60 ℃搅拌回流72 h。反应结束后，向体系中加入30 mL去离子水，分液得有机相，再用30 mL CHCl_3_对水相进行3次重复萃取，合并所有收集的有机相。向收集的有机相中加入无水MgSO_4_进行干燥处理，抽滤，滤液旋蒸除去溶剂，所得的固体用正己烷反复洗涤数次，最后在60 ℃真空条件下干燥12 h，即得到烯基功能化修饰的手性Pim产物。

**图2:**
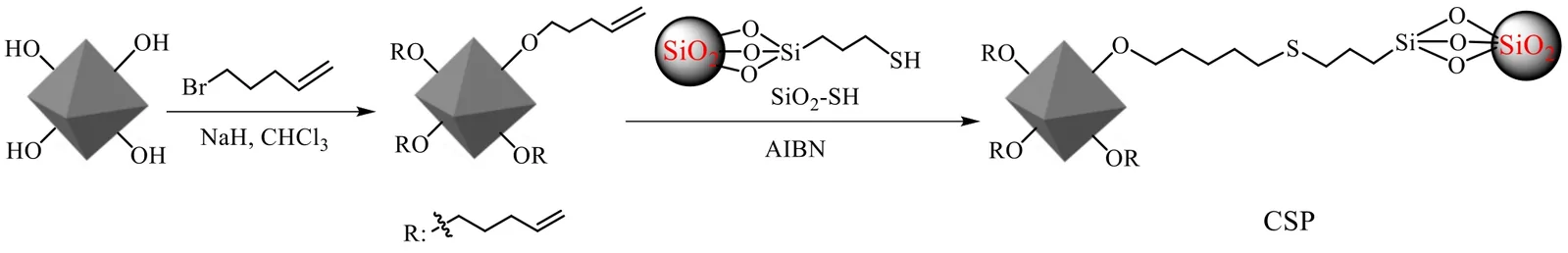
CSP的制备

同样在N_2_氛围下，向100 mL圆底烧瓶中加入上述制得的烯基功能化修饰的手性Pim（0.5 g）、SiO_2_-SH（0.98 g）、AIBN（0.08 g）和50 mL MeOH，缓慢升温至60 ℃搅拌回流反应3天。反应结束后，抽滤，得到的固体产物依次用MeOH和丙酮进行多次洗涤，然后在100 ℃真空干燥箱中干燥18 h，得到CSP。

#### 1.2.3 色谱柱的填充和HPLC条件

称取1.2 g制备的CSP置于烧杯中，加入30 mL *n*-HEX-2-PrOH（90:10，体积比），通过超声处理使其均匀分散在溶剂中制得匀浆液。以相同配比的溶液作为推进溶剂，在50 MPa的高压条件下将上述匀浆液均匀压入HPLC空柱内。维持该压力5 min后，将系统压力调节至25 MPa，恒压保持30 min，即完成色谱柱的填充。将填充完成的色谱柱接入HPLC分析系统，以*n*-HEX-2-PrOH（90:10，体积比）为流动相冲洗至基线平稳。

HPLC测试条件：NP-HPLC分离模式下，选用不同体积比的*n*-HEX-2-PrOH作为流动相；RP-HPLC分离模式则采用不同体积比的MeOH-H_2_O作为流动相；两种模式下均设定流速为0.1 mL/min，检测波长254 nm，柱温控制为25 ℃。

## 2 结果与讨论

### 2.1 四醛基化合物1、手性Pim和CSP的表征

采用NMR、FT-IR、ESI-MS、TGA、EA等方法对合成的四醛基化合物1、手性Pim及CSP进行了表征。其中，四醛基化合物1的NMR数据如下：^1^H NMR （500 MHz，CDCl_3_） *δ*： 11.17（s，OH，2H）， 10.22（s，CHO，4H）， 8.05（d，ArH，1H）， 7.95（s，ArH，4H）， 7.84（td，ArH，1H）， 7.69（td，ArH，1H）， 7.64（dt，ArH，1H）； ^13^C NMR（125 MHz，CDCl_3_） *δ*：168.37， 163.74， 149.82， 135.38， 132.30， 130.59， 126.93， 125.18， 123.57， 88.88， 53.29， 20.44（谱图见附图S1，https：//www.chrom-China.com）。Pim的ESI-MS图（图3）中，*m/z* 1 173.525 5和*m/z* 587.264 7处的特征信号分别对应Pim的［M+H］^+^和［M+2H］^2+^离子峰，这与Pim分子式C_72_H_68_N_8_O_8_的理论计算值（［M+H］⁺=［C_72_H_68_N_8_O_8_+H］⁺，*m/z* 1 173.523 8；［M+2H］^2+^=［C_72_H_68_N_8_O_8_+2H］^2+^，*m/z* 587.265 9）相吻合。在Pim的FT-IR谱图（图4a）中，3 423 cm^-1^处的宽吸收峰是-OH的伸缩振动，2 930 cm^-1^和2 857 cm^-1^处的吸收峰为-CH_2_-和-CH-的伸缩振动，1 766 cm^-1^和1 636 cm^-1^处的吸收峰分别为-C=O和-C=N-的伸缩振动特征峰，1 522、1 461和1 364 cm^-1^处为苯环骨架振动吸收峰。此外，Pim的^1^H NMR谱图（见附图S2）数据也与文献报道一致。上述系列表征结果证实Pim已成功合成。

**图3:**
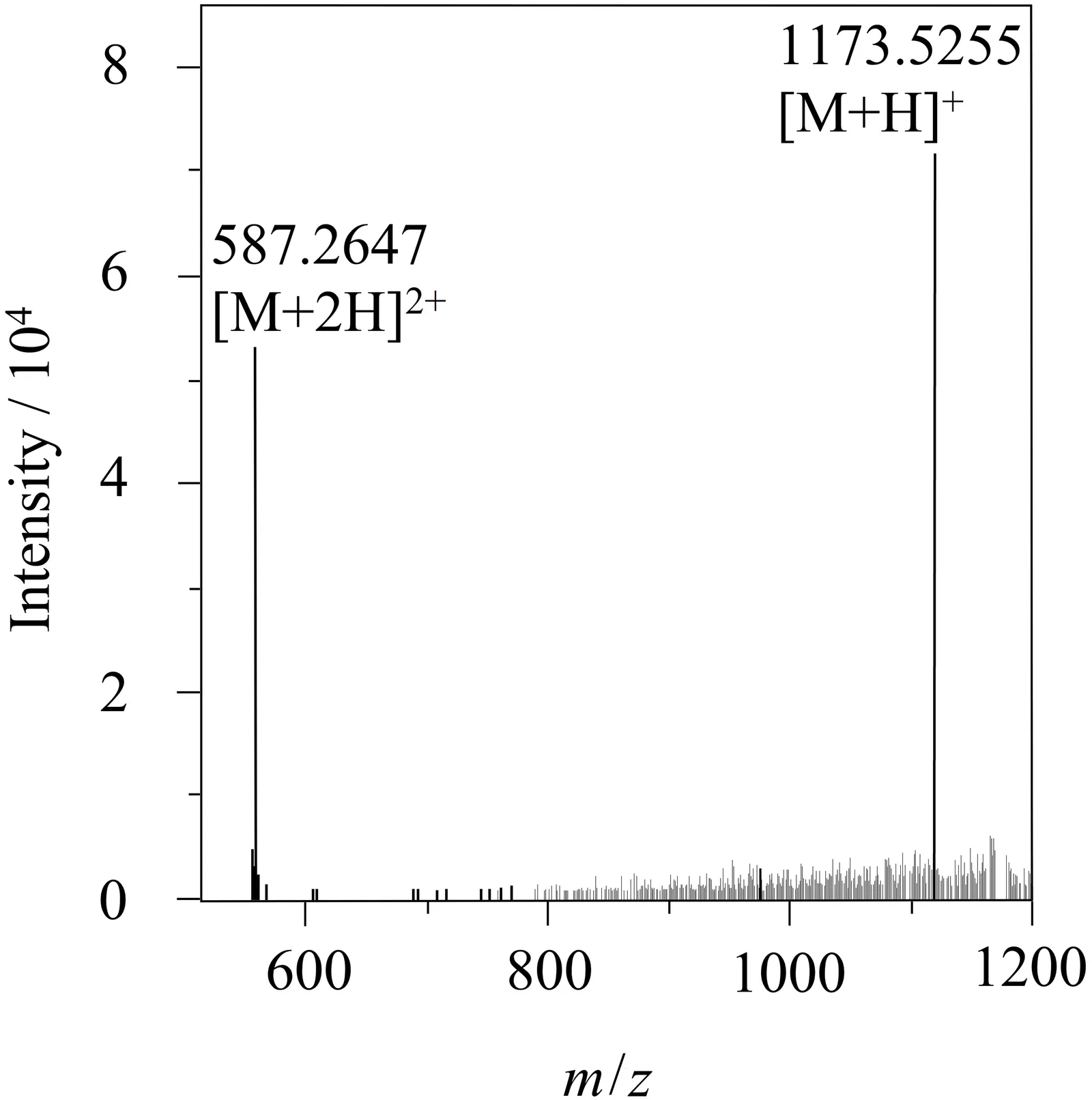
Pim的ESI-MS图

**图4:**
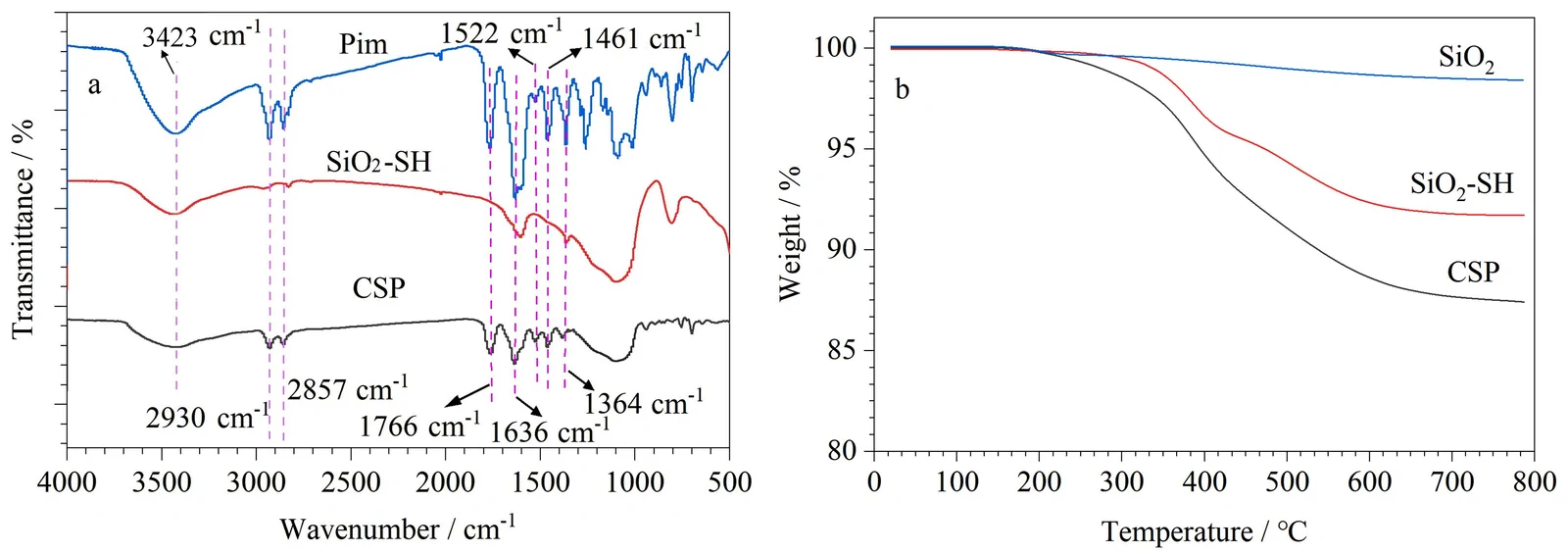
（a）Pim、SiO_2_-SH和CSP的FT-IR图；（b）SiO_2_、SiO_2_-SH和CSP的TGA图

Pim、SiO_2_-SH和CSP的FT-IR光谱对比图如图4a所示。从图中可观察到，与SiO_2_-SH相比，CSP在1 766、1 636、1 522、1 461和1 364 cm^-1^处出现了新的特征吸收峰，并且在2 930 cm^-1^和2 857 cm^-1^处吸收峰显著增强，表明Pim已成功键合在SiO_2_-SH上。由Pim、SiO_2_-SH和CSP的TGA对比曲线可以看出（图4b），CSP的质量损失显著高于SiO_2_-SH，这是由于CSP上键合的有机组分Pim的分解所致。同时表1中元素分析结果显示CSP中C、H和N 3种元素的含量均明显高于SiO_2_-SH，这些结果均证实了Pim成功键合在SiO_2_-SH上。根据表面键合量计算公式^［39］^，计算得到CSP表面键合量为0.41 μmol/m^2^。为了测定该CSP填充制备的Pim柱的柱效，以*n*-HEX-2-PrOH（90:10，体积比）为流动相的NP-HPLC分离模式下，用苯为分析物测得其理论塔板数为14 200块/m；以MeOH-H_2_O（80:20，体积比）为流动相的RP-HPLC分离模式下，用萘为分析物测得其理论塔板数为15 500块/m。

**表1:** SiO_2_-SH和CSP的元素分析数据

Sample	Contents/%		
	C	H	N
SiO_2_-SH	4.791	0.305	<0.05%
CSP	18.458	1.941	1.920

### 2.2 Pim柱在NP-HPLC模式下对外消旋体的拆分

为了评估所制备的Pim柱在NP-HPLC分离模式下的对映体拆分性能，以*n*-HEX-2-PrOH（90:10，体积比）为流动相，对包括醇、胺、酯、环氧化合物等在内的多种外消旋体进行了拆分实验。结果显示，在该分离模式下，Pim柱对10种外消旋体（分子结构见附图S3）具有良好的手性拆分效果，它们的拆分色谱图如图5所示，相关的色谱性能评价参数见表2。由图5和表2可知，这10种外消旋体中4-氟-*α*-甲基苄胺、氧化苯乙烯、扁桃酸乙酯、安息香和4-甲基二苯基甲醇这5种得到了基线分离（分离度*R*_s_>1.5）。为凸显所制备的CSP的潜在价值，上述被拆分的10种外消旋体也在两种常用商品手性柱（Chiralpak AD-H柱和Chiralcel OD-H柱）上进行了拆分测试，3种色谱柱对这10种外消旋体拆分的对比色谱数据列于表2中，对比色谱图见附图S4。因为Pim柱的内径（2.1 mm）与Chiralpak AD-H柱和Chiralcel OD-H柱的内径（4.6 mm）不同，所以它们所使用的流速也不相同，均采用它们的较优的流速。分析对比结果发现，Chiralpak AD-H柱无法拆分4-氟-*α*-甲基苄胺、1-苯基-1-戊醇和1-（4-氟苯基）乙醇这3种外消旋体，但它们在Pim柱上可获得拆分；Chiralcel OD-H柱未能对氧化苯乙烯、1-苯基-1-戊醇和1-（4-氟苯基）乙醇这3种外消旋体实现拆分，而Pim柱可拆分这3种外消旋体。另外，一些外消旋体在Pim柱上拆分的*R*_s_值也高于其在Chiralpak AD-H柱或Chiralcel OD-H柱上拆分的*R*_s_值。例如，氧化苯乙烯、扁桃酸乙酯、安息香和4-甲基二苯基甲醇在Pim柱上拆分的*R*_s_值高于它们在Chiralpak AD-H柱上拆分的*R*_s_值；4-氟-*α*-甲基苄胺、2-甲氧基-2-苯基乙醇、安息香和4-甲基二苯基甲醇在Pim柱上拆分的*R*_s_值也高于它们在Chiralcel OD-H柱上拆分的*R*_s_值。综上可得，该手性Pim柱的手性拆分性能与这两种商品手性柱具有良好的互补效应，能拆分这两种商品柱不能拆分或拆分效果不理想的一些手性化合物，具有潜在的应用价值。

**图5:**
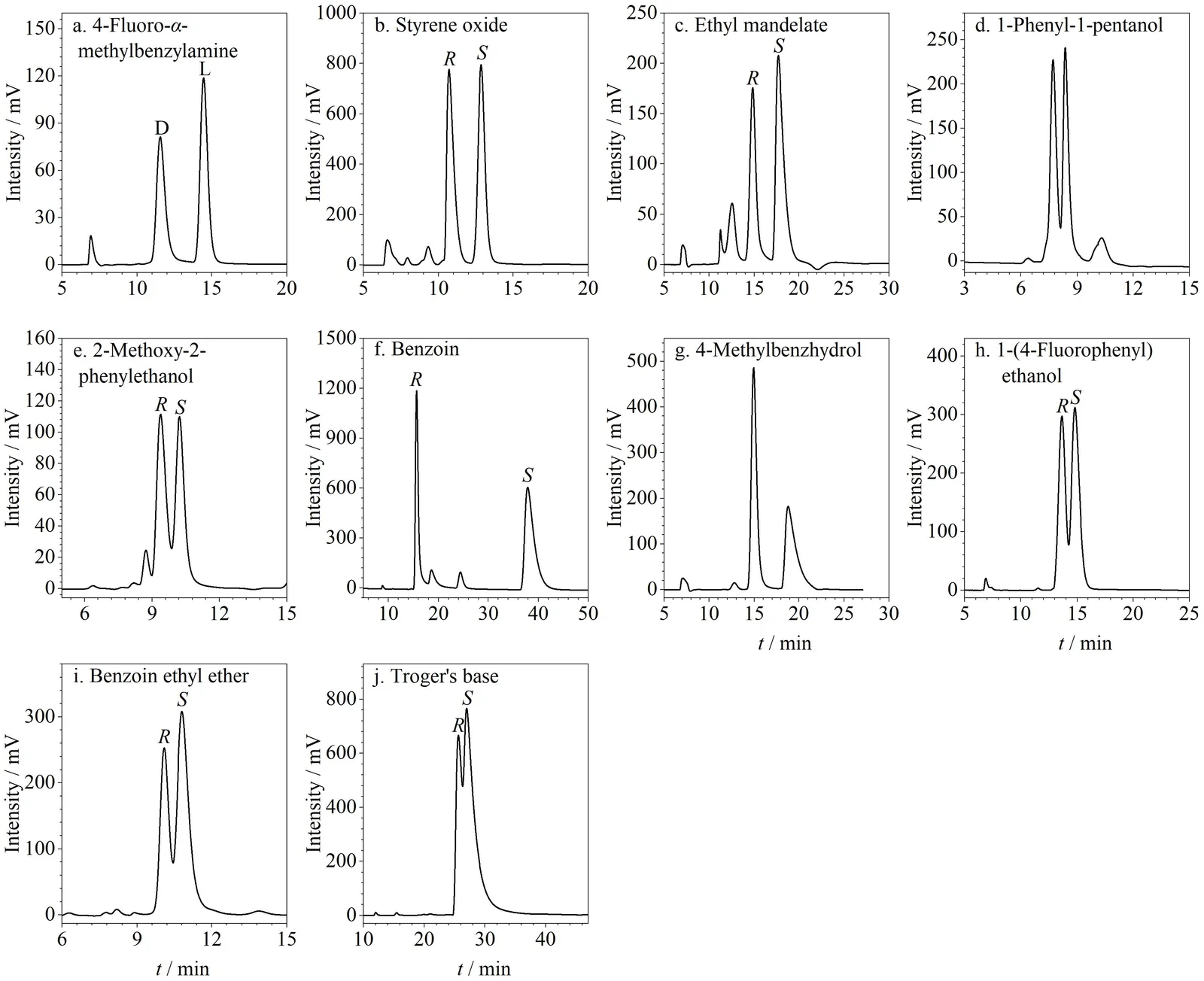
NP-HPLC分离模式下外消旋体在Pim柱上的色谱图

**表2:** 在NP-HPLC分离模式下外消旋体在Pim、Chiralpak AD-H和Chiralcel OD-H柱上的分离数据

Racemate	Pim				Chiralpak AD-H				Chiralcel OD-H		
	*k*_1_	*α*	*R*_s_		*k*_1_	*α*	*R*_s_		*k*_1_	*α*	*R*_s_
4-Fluoro-*α*-methylbenzylamine	0.75	1.53	2.64		1.62	1.00	-		0.93	1.13	0.73
Styrene oxide	0.63	1.52	2.48		0.41	1.10	0.50		0.64	1.00	-
Ethyl mandelate	1.26	1.34	1.95		1.92	1.08	1.63		1.45	2.04	7.68
1-Phenyl-1-pentanol	0.17	1.57	1.12		0.78	1.00	-		0.74	1.00	-
2-Methoxy-2-phenylethanol	0.43	1.30	1.14		0.91	1.23	2.70		0.81	1.13	0.69
Benzoin	1.38	3.45	10.35		4.54	1.34	6.35		2.52	1.58	6.53
4-Methylbenzhydrol	1.27	1.46	2.44		1.66	1.11	1.48		1.74	1.14	1.27
1-（4-Fluorophenyl）ethanol	1.08	1.17	0.96		0.83	1.00	-		0.74	1.00	-
Benzoin ethyl ether	0.53	1.20	0.92		0.82	1.17	2.31		0.39	1.17	1.52
Troger’s base	2.90	1.07	0.53		0.66	1.83	6.99		1.07	1.26	1.71

Mobile phase： *n*-HEX-2-PrOH （90:10， volume ratio） for the three columns. Flow rates： 0.1 mL/min for the Pim column， and 0.5 mL/min for Chiralpak AD-H and Chiralcel OD-H columns. NP-HPLC： normal-phase high performance liquid chromatography； *k*_1_： retention factor of the first enantiomer； *α*： separation factor； *R*_s_： resolution value； -： no separation.

### 2.3 Pim柱在RP-HPLC模式下对外消旋体的拆分

以甲醇-水（MeOH-H_2_O）作为流动相，我们也评价了Pim柱在RP-HPLC分离模式下的手性拆分性能。实验结果表明，Pim柱在RP-HPLC分离模式下对包括醇类、酮类、含氮杂环、环氧化合物等不同类型的9种外消旋体（分子结构见附图S5）具较好的手性拆分效果。由表3中的分离数据和图6的色谱图可知，这9种外消旋体中有8种在Pim柱上获得了基线分离。通常情况下，Chiralpak AD-H和Chiralcel OD-H柱在正相分离模式下的对映选择性优于反相分离模式。因此，我们也将这9种外消旋体在Chiralpak AD-H和Chiralcel OD-H柱上在NP-HPLC分离模式下进行了分离测试，所得的分离结果与Pim柱进行了对比，表3中列出了它们分离的对比色谱数据，附图S6是它们的分离对比色谱图。对比结果显示，Chiralpak AD-H柱未能拆分1-（4-氟苯基）乙醇、1-苯基-1-戊醇、1-苯基乙醇和佐匹克隆这4种外消旋体，Chiralcel OD-H柱未能拆分1-（4-氟苯基）乙醇、1-苯基-1-戊醇、佐匹克隆、4-氯二苯甲醇和氧化苯乙烯这5种外消旋体。更有意义的是，Pim柱可拆分这两种商品柱均不能拆分的1-（4-氟苯基）乙醇、1-苯基-1-戊醇和佐匹克隆这3种外消旋体。此外，部分外消旋体在Pim柱上的分离效果也优于这两种商品柱。例如，4，4′-二甲基安息香和氧化苯乙烯在Pim柱上拆分的*R*_s_值高于其在Chiralpak AD-H柱上拆分的*R*_s_值；4，4′-二甲基安息香、1-苯基乙醇和2-甲氧基-2-苯乙醇在Pim柱上拆分的*R*_s_值也高于其在Chiralcel OD-H柱上拆分的*R*_s_值。以上实验结果充分表明Pim柱在NP-HPLC和RP-HPLC分离模式下均具有良好的手性分离性能，还与Chiralpak AD-H和Chiralcel OD-H两种商品手性柱形成良好的手性拆分互补，表明Pim材料在HPLC手性分离领域具有良好的研究意义和价值。

**表3:** RP-HPLC分离模式下外消旋体在Pim、Chiralpak AD-H和Chiralcel OD-H柱上的分离数据

Racemate	Pim				Chiralpak AD-H				Chiralcel OD-H		
	*k*_1_	*α*	*R*_s_		*k*_1_	*α*	*R*_s_		*k*_1_	*α*	*R*_s_
4，4′-Dimethylbenzoin	0.99	1.66	2.87		5.06	1.10	2.74		1.69	1.36	2.39
1-（4-Fluorophenyl）ethanol	0.57	1.35	1.36		0.83	1.00	-		0.74	1.00	-
1-Phenyl-1-pentanol	0.82	1.57	2.90		0.78	1.00	-		0.74	1.00	-
1-Phenylethanol	0.60	1.40	1.79		0.84	1.00	-		0.90	1.22	1.29
Zopiclone	0.90	2.73	9.46		1.71	1.00	-		2.09	1.00	-
4-Chlorobenzhydrol	0.59	1.38	1.77		1.65	1.14	2.01		2.05	1.00	-
Styrene oxide	1.19	1.40	2.60		0.41	1.10	0.50		0.64	1.00	-
2-Methoxy-2-phenylethanol	1.71	1.26	2.41		0.91	1.23	2.70		0.81	1.13	0.69
Benzoin	0.83	1.49	1.91		4.54	1.34	6.35		2.52	1.58	6.53

Mobile phases： MeOH-H_2_O （80:20， volume ratio） for Pim column； *n*-HEX-IPA （90:10， volume ratio） for Chiralpak AD-H and Chiralcel OD-H columns. Flow rates： 0.1 mL/min for the Pim column， and 0.5 mL/min for Chiralpak AD-H and Chiralcel OD-H columns. RP： reversed-phase.

**图6:**
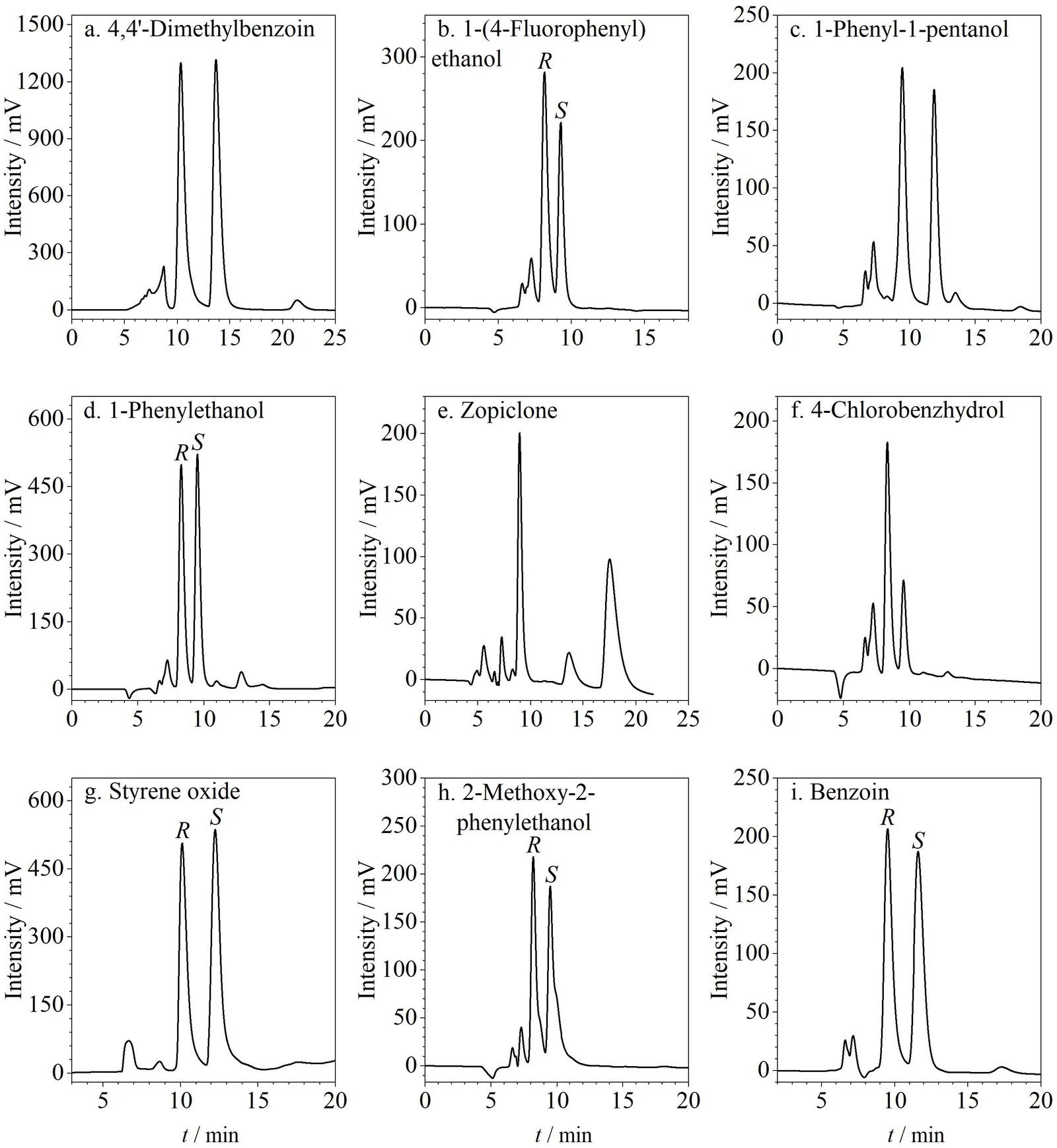
在RP-HPLC分离模式下外消旋体在Pim柱上的色谱图

### 2.4 柱温对手性拆分的影响

柱温是影响色谱分离效果的重要因素之一。为研究柱温对Pim柱手性拆分效果的影响，本研究以4-氟-*α*-甲基苄胺和安息香为目标分析物，在25~45 ℃范围内进行手性拆分试验（图7a、7c）。随着柱温逐步升高至45 ℃，4-氟-*α*-甲基苄胺和安息香在Pim柱上的保留时间逐渐缩短，*R*_s_值也相应减小（见附图S7），这一现象表明这两种对映体在Pim柱上的手性分离过程属于放热过程。进一步对两种分析物进行van’t Hoff方程拟合分析，其van’t Hoff曲线（图7b、7d）中1/*T*与ln *k*呈现良好的线性关系，证明在此温度区间内Pim柱的手性分离机制未发生改变。计算得到的相关热力学数据列于表4。由表4可知，两种分析物对映体的Δ*G*值均为负值，说明分析物从流动相向CSP转移过程具有热力学自发性。从理论来讲，Δ*G*绝对值越大，分析物越易与CSP发生相互作用并转移至其表面，对应保留时间越长。L-4-氟-*α*-甲基苄胺的Δ*G*绝对值比D-4-氟-*α*-甲基苄胺的更大，*S*-安息香的Δ*G*绝对值大于*R*-安息香的，该热力学规律与4-氟-*α*-甲基苄胺和安息香对映体在Pim柱上的实际洗脱顺序完全吻合。

**图7:**
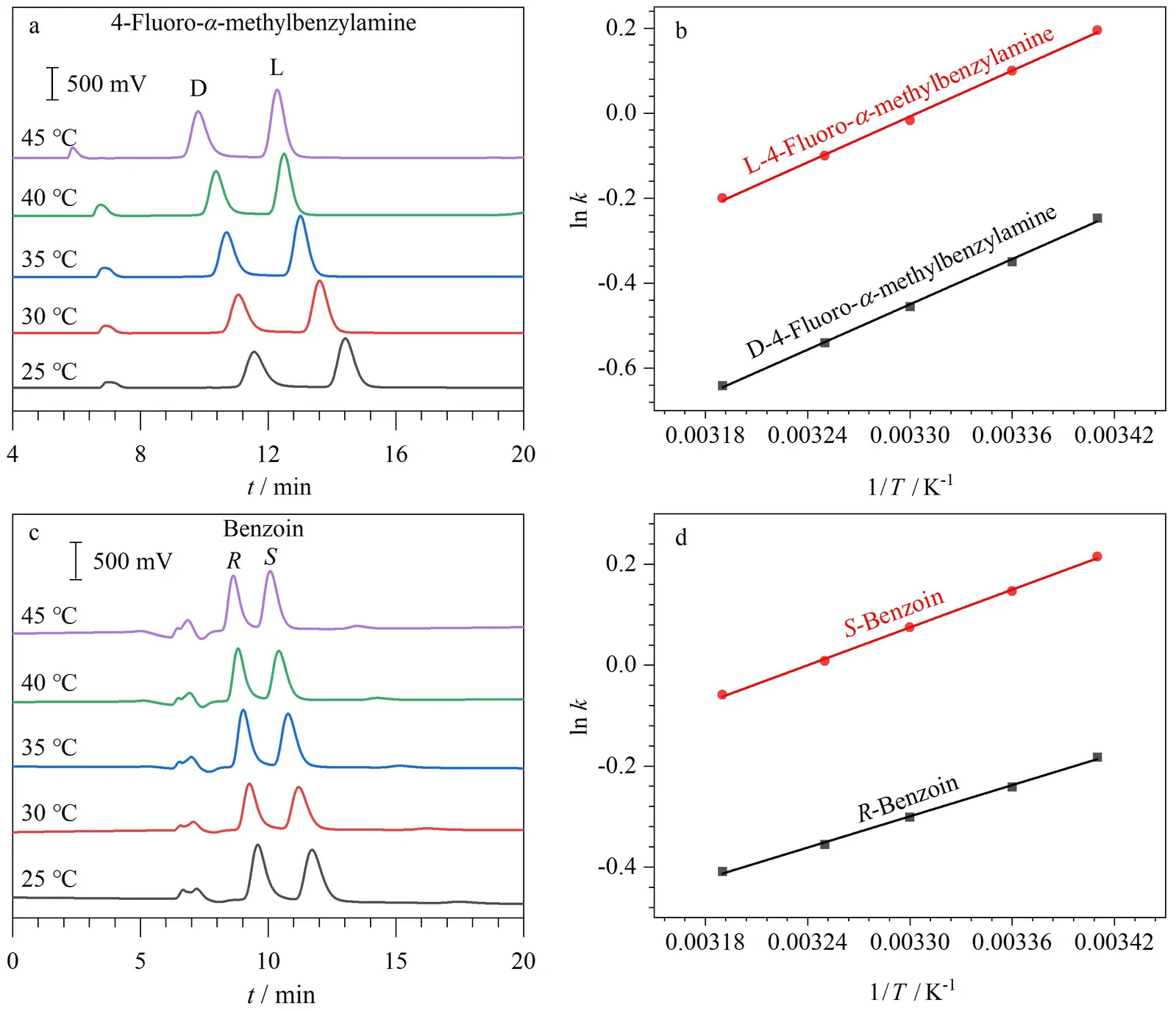
柱温对Pim柱手性分离效果的影响

**表4:** NP-HPLC下拆分4-氟- ***α*** -甲基苄胺和RP-HPLC下拆分安息香的热力学数值

Analyte	*ΔH/*（kJ/mol）	*ΔS/*（J/（mol·K））	*ΔG/*（kJ/mol）	*R* ^2^
D-4-Fluoro-*α*-methylbenzylamine	-14.77±0.33	-45.94±1.10	-1.08±0.33	0.9980
L-4-Fluoro-*α*-methylbenzylamine	-14.96±0.33	-42.89±1.10	-2.17±0.33	0.9980
*R*-Benzoin	-8.58±0.22	-24.24±0.71	-1.35±0.22	0.9975
*S*-Benzoin	-10.38±0.19	-27.07±0.63	-2.31±0.19	0.9987

### 2.5 进样量对手性拆分的影响

为了研究分析物进样量对Pim柱手性拆分效果的影响，我们选取4-氟-*α*-甲基苄胺和安息香作为分析物，在柱温为25 ℃，将进样量从1 μg逐步增加至20 μg，所得的分离色谱图如图8a、8c所示。在进样量增加过程中，两种分析物在Pim柱上分离的保留时间未发生明显改变，手性拆分效果也基本保持稳定，分析物色谱峰面积与进样量之间呈现出良好的线性递增关系（图8b、8d）。当进样量增加到20 μg时，Pim柱对这两种分析物仍可实现较好的分离。色谱柱的柱容量对其实际应用具有重要的意义，对于不同的分析物，色谱柱对其分离的柱容量会有不同。Pim柱在NP-HPLC分离模式下对4-氟-*α*-甲基苄胺的柱容量约为50 μg，在RP-HPLC分离模式下对安息香的柱容量约为30 μg。因此，Pim柱不仅在一定进样量区间内具备稳定的手性拆分效能，还拥有良好的柱容量和定量分析特性。

**图8:**
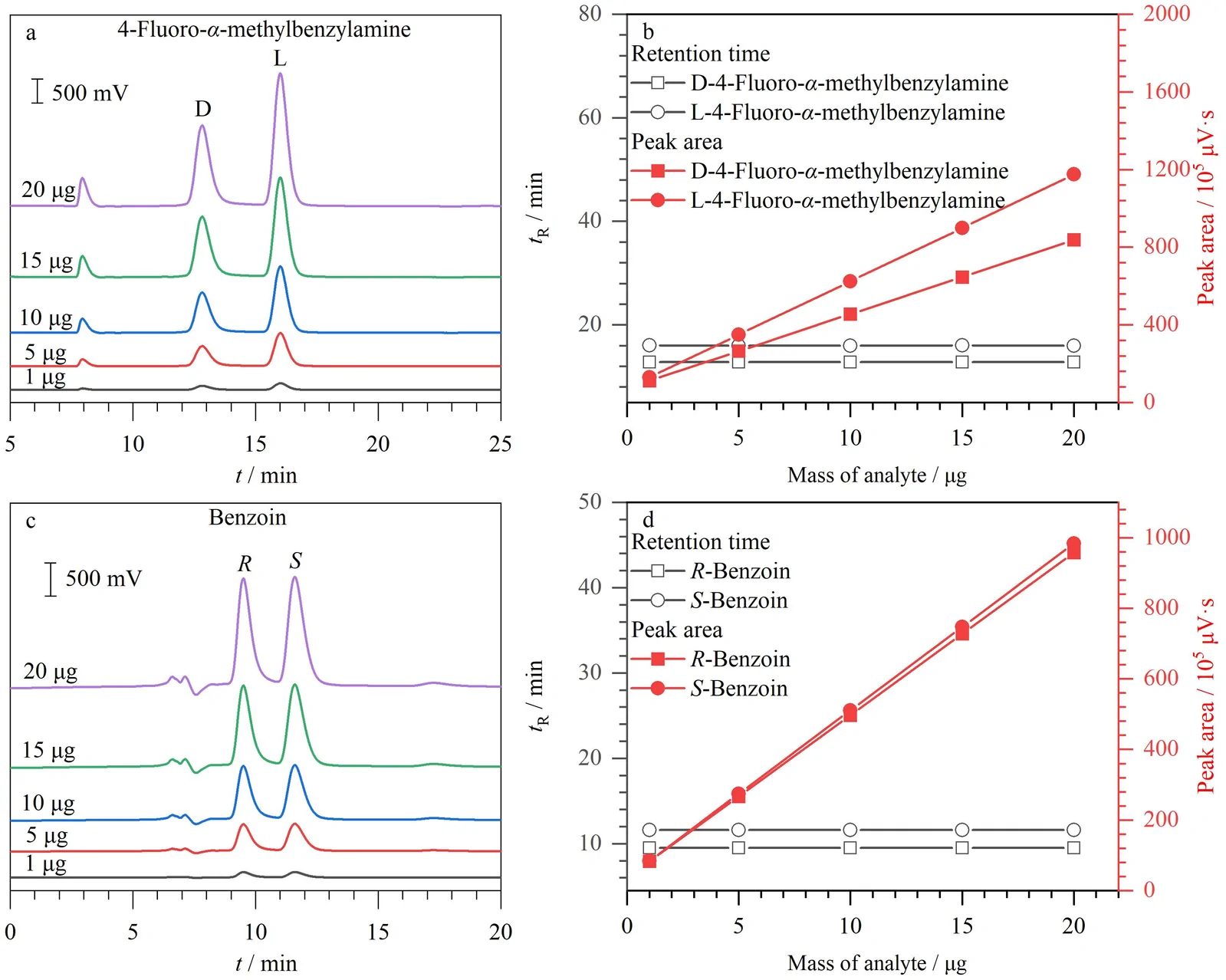
进样量对Pim柱手性分离效果的影响

### 2.6 柱的重复性和稳定性

为评价Pim柱的重复性和稳定性，我们以4-氟-*α*-甲基苄胺和安息香为目标分析物，在柱历经不同次数进样使用后再次对其进行分离测试。实验结果显示，在柱历经100、200、300和400次进样使用后对这两种分析物分离的保留时间和分离度与柱使用之初对它们的分离效果相比无明显变化（图9），其分离的保留时间和分离度的相对标准偏差（RSD，*n*=5）分别低于1.50%和1.92%。在柱放置保存1个月后，其分离效果与柱使用之初的分离效果也无显著改变，对这两种分析物分离的保留时间和分离度的RSD（*n*=3）分别低于1.88%和2.26%。以上结果表明Pim柱具有较好的分离重复性和稳定性。

**图9:**
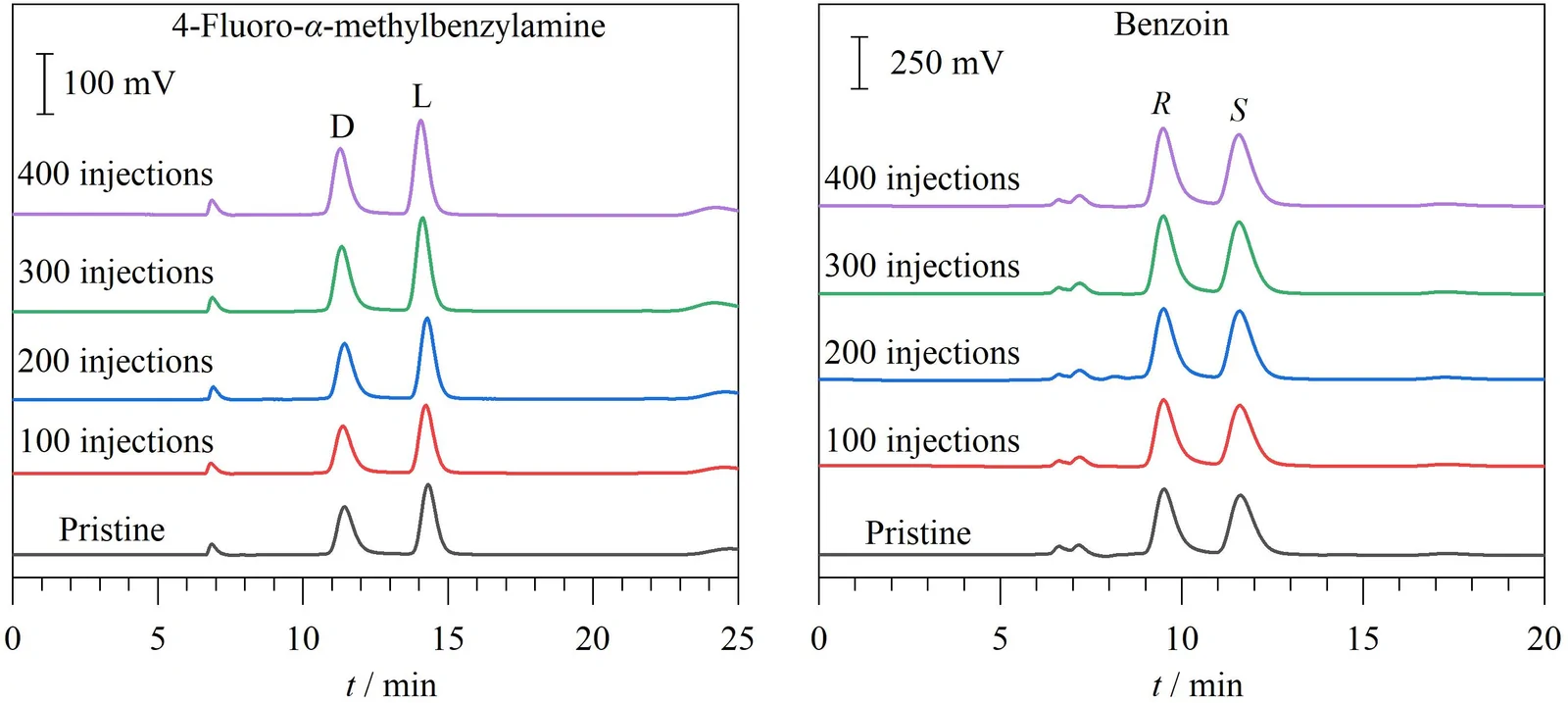
Pim柱分离的重复性和稳定性

## 3 结论

本文将合成的一种［2+4］型手性POC Pim作为手性选择剂，通过巯基-烯点击反应将其键合到巯基硅胶基质表面制备了高效液相色谱CSP，并评价了其在NP-HPLC和RP-HPLC分离模式下的手性拆分性能。结果表明，所制备的Pim柱展现出良好的手性拆分性能，在NP-HPLC分离模式下有10种外消旋体获得了拆分，RP-HPLC分离模式下有9种外消旋体获得了拆分，并且与Chiralcel OD-H和Chiralpak AD-H两种商品手性柱有良好的手性拆分互补性，能拆分这两种商品柱无法拆分或拆分效果不理想的一些外消旋体。此外，该Pim柱在历经数百次进样使用和一段时间放置保存后仍具有较好的分离重复性和稳定性。本研究不仅证实了手性POC Pim是一种良好的手性分离材料，也为POCs类新型高效液相色谱CSP的研究提供了重要参考。

## References

[1] Huang B， Li K， Ma Q Y， et al . Anal Chem， 2023， 95（35）： 13289 10.1021/acs.analchem.3c02438 37615071

[2] Qian H L， Xu S T， Yan X P . Anal Chem， 2023， 95： 304 10.1021/acs.analchem.2c04371 36625130

[3] Li H， Wang X P， Shi C X， et al . Chin Chem Lett， 2023， 34（3）： 107606

[4] Zhang J H， Xie S M， Yuan L M . J Sep Sci， 2022， 45（1）： 51 10.1002/jssc.202100593 34729907

[5] Zhang J H， Liang R X， Huang B， et al . Chin Chem Lett， 2026， 37（1）： 111146

[6] Sun G， Luo Y， Yan Z， et al . Chin Chem Lett， 2024， 35（12）： 109787

[7] Zheng D S， Tang W Q， Zhu J P， et al . Chinese Journal of Chromatography， 2024， 42（6）： 524 10.3724/SP.J.1123.2024.01022 PMC11165387 38845513

[8] Yang M F， Zheng K N， Long Y X， et al . Chinese Journal of Chromatography， 2025， 43（4）： 335 10.3724/SP.J.1123.2024.06004 PMC11966380 40133199

[9] Tang W Q， Cheng Y， Zhu J P， et al . Angew Chem Int Ed， 2024， 63（42）： e202409588 10.1002/anie.202409588 39060222

[10] Qian H L， Yang C X， Yan X P . Nat Commun， 2016， 7： 12104 10.1038/ncomms12104 PMC4945876 27401541

[11] Tang B， Wang W， Hou H， et al . Chin Chem Lett， 2022， 33（2）： 898

[12] Zhou S Q， Kuang Y X， Zheng J， et al . Chinese Journal of Chromatography， 2024， 42（1）： 1 10.3724/SP.J.1123.2023.07021 PMC10782275 38197202

[13] Yuan C， Wang Z， Xiong W Q， et al . J Am Chem Soc， 2023， 145（34）： 18956 10.1021/jacs.3c05973 37596711

[14] Li X， Cui Y Y， Yang C X . Talanta， 2021， 224： 121914 10.1016/j.talanta.2020.121914 33379116

[15] Tozawa T， Jones J T A， Swamy S I， et al . Nat Mater， 2009， 8（12）： 973 10.1038/nmat2545 19855385

[16] Mastalerz M . Angew Chem Int Ed， 2010， 49（30）： 5042 10.1002/anie.201000443 20572217

[17] Yang X C， Ullah Z， Stoddart J F， et al . Chem Rev， 2023， 123： 4602 10.1021/acs.chemrev.2c00667 PMC10141292 37023354

[18] Wang H， Jin Y， Sun N， et al . Chem Soc Rev， 2021， 50（16）： 8874 10.1039/d0cs01142h 34180920

[19] Kong Q D， Liu L L， Li Z Y . Chem Eur J， 2024， 30： e202400947 10.1002/chem.202400947 38622630

[20] Cao X， Jin Y， Wang H， et al . Chin Chem Lett， 2024， 35（9）： 109201

[21] Zhao X， Cui H Y， Guo L L， et al . Angew Chem Int Ed， 2024， 63（47）： e202411613 10.1002/anie.202411613 39140458

[22] Mohan M， Pham D-J， Fluck A， et al . Chem Eur J， 2024， 30： e202400458 10.1002/chem.202400458 38427204

[23] Zhang Z Y， Ying Y， Li Y F， et al . Angew Chem Int Ed， 2026， 65（3）： e22364 10.1002/anie.202522364 41287360

[24] Guo Z X， Kong W X， Zhou L Y， et al . ACS Catal， 2025， 15： 3955

[25] Xu Y， Dai C， Xu Z F . Talanta， 2025， 286： 127518 10.1016/j.talanta.2025.127518 39755078

[26] Kelabi D A， Dey A， Alimi L O， et al . Chem Sci， 2022， 13： 7341 10.1039/d2sc00836j PMC9214840 35799823

[27] Zhang J H， Xie S M， Chen L， et al . Anal Chem， 2015， 87（15）： 7817 10.1021/acs.analchem.5b01512 26145712

[28] Xie S M， Zhang J H， Fu N， et al . Anal Chim Acta， 2016， 903： 156 10.1016/j.aca.2015.11.030 26709309

[29] Zhang J H， Xie S M， Wang B J， et al . J Chromatogr A， 2015， 1426： 174 10.1016/j.chroma.2015.11.038 26632517

[30] Wang Y， Li H X， Xie S M， et al . Microchem J， 2021， 170： 106650

[31] Huang B， Chen J， Wang B J， et al . Chinese Journal of Chromatography， 2024， 42（9）： 891 10.3724/SP.J.1123.2024.01025 PMC11358875 39198948

[32] Wang Y， Chen J K， Xiong L X， et al . Anal Chem， 2022， 94（12）： 4961 10.1021/acs.analchem.1c03626 35306818

[33] Zhang H C， Zhang J H， Gong Y N， et al . Chem Eur J， 2025， 31（31）： e202500977

[34] Liang R X， Zhang Y P， Zhang J H， et al . J Chromatogr A， 2023， 1711： 464444 10.1016/j.chroma.2023.464444 37837712

[35] Li Z， Tang Z， Cao J， et al . J Chromatogr A， 2024， 1737： 465441 10.1016/j.chroma.2024.465441 39427509

[36] Jiang Z， Zhang G， Yang Y， et al . J Chromatogr A， 2025， 1740： 465561 10.1016/j.chroma.2024.465561 39626335

[37] Zhang L， Jia Y， Meng F S， et al . Dyes Pigments， 2022， 206： 110578

[38] Yao X， Tan T T， Wang Y . J Chromatogr A， 2014， 1326： 80 10.1016/j.chroma.2013.12.054 24411138

[39] Siles B A， Halsall H B， Dorsey J G . J Chromatogr A， 1995， 704： 289 10.1016/0021-9673(95)00269-s 7670627

